# Patterns of the initiation of disease-modifying antirheumatic drugs in incident rheumatoid arthritis: a German perspective based on nationwide ambulatory drug prescription data

**DOI:** 10.1007/s00296-018-4161-7

**Published:** 2018-10-10

**Authors:** Annika Steffen, Jakob Holstiege, Kerstin Klimke, Manas K. Akmatov, Jörg Bätzing

**Affiliations:** Central Research Institute of Ambulatory Health Care in Germany (Zi), Salzufer 8, 10587 Berlin, Germany

**Keywords:** Ambulatory drug prescription data, Biologicals, Disease-modifying antirheumatic drugs, Glucocorticoids, Non-steroidal anti-inflammatory drugs, Rheumatoid arthritis

## Abstract

This study aimed at providing a current and nearly complete picture of the patterns of the initiation of disease-modifying antirheumatic drugs (DMARDs) in patients with newly diagnosed RA. Based on ambulatory drug prescription data and physician billing claims data covering 87% of the German population, we assembled a cohort of incident RA patients aged 15–79 years (*n* = 54,896) and assessed the prescription frequency of total DMARDs, conventional synthetic (csDMARDs) and biologic DMARDs (bDMARDs) within the first year of disease. Using multiple logistic regression, we estimated the chance of early DMARD receipt based on age, sex, serotype and specialty of prescribing physician while controlling for region of residence. In total, 44% of incident RA patients received a DMARD prescription within the first year of disease. In multiple regression, younger patients (< 35 years) had 1.7-fold higher chances of receiving a csDMARD than patients aged ≥ 65 years [odds ratio (OR): 1.65 with 95% confidence interval (CI) 1.51–1.80] and almost tenfold higher chances to receive a bDMARD [OR (95% CI) 9.5 (8.0–11.3)]. Seropositivity and a visit to a rheumatologist were positively associated with DMARD initiation [OR (95% CI) 2.8 (2.6–2.9) and 5.9 (5.6–6.2) for csDMARDs, respectively]. Based on data covering 87% of the German population, the present study revealed that less than half of incident RA patients receive DMARDs within the first year of disease and that marked differences exist according to age. The study highlights the importance of involving a rheumatologist early in the management of RA.

## Background

Rheumatoid arthritis (RA) is a chronic autoimmune disease characterized by inflammation of synovial tissues that leads to progressive, irreversible joint destruction, impaired joint function and pain [1]. It is one of the most prevalent chronic inflammatory diseases, affecting approximately 1% of the adult population in developed nations worldwide [2]. On the basis of nationwide claims data, we recently estimated the RA prevalence to be 1.2% in the adult population in Germany and the incidence amounting to 80 per 100,000 persons which corresponds to roughly 50,000 new cases in Germany per year [3]. On an individual level, RA is related to reduced quality of life and increased co-morbidity, including premature mortality [4]. On a societal level, RA imposes a high economic burden due to healthcare costs and work productivity loss.

With the advent of highly effective medications, the management of RA has changed profoundly over the past 25 years [5]. RA management has evolved from a strategy of providing symptomatic relief to therapeutic regimens modifying disease activity, thereby preventing disease progression and joint destruction and improving health-related quality of life. Modern RA management focusses on early diagnosis, immediate initiation of effective therapy with disease-modifying antirheumatic drugs (DMARDs) and the requirement to set a therapeutic target (‘treat-to-target strategy’, T2T) [6–11]. Aggressive treatment during the early phase of RA has been shown to be more likely to succeed in preventing long-term sequelae and preserving functional status compared to the same treatment applied at later stages of the disease. This therapeutic ‘window-of-opportunity’ is widely accepted and refers to the timely initiation of DMARD therapy, ideally within the first 3 months of disease onset. Regular monitoring of the disease activity and adjustments of the therapy to the target of sustained remission (or low disease activity if remission is unattainable) are important aspects of the T2T strategy consistently embedded into national and international recommendations [6–11].

DMARDs, as the central component of every modern medication-based RA treatment regimen, are divided into two major classes: conventional synthetic (csDMARDs) and biologic DMARDs (bDMARDs). In addition, pharmacologic treatment of RA may include short-term glucocorticoids (GCs) as a bridging therapy [6] and non-steroidal anti-inflammatory drugs (NSAIDs) for pain relief.

Despite the importance of immediate DMARD therapy for virtually all patients with incident (active) RA, studies from the US, Canada and the UK suggest a considerable underuse of DMARDs among patients with newly diagnosed RA, indicating that only 50–60% of incident cases receive DMARDs within 1 year of diagnosis [12–14]. For Germany, it is currently unknown how well guideline recommendations are reflected in routine clinical care of the ambulatory setting. Although data on the treatment of RA patients within the specialty care of the German Collaborative Arthritis Centers do exist [15], data on the real-world treatment with DMARDs are limited to a study based on prevalent cases [16].

The present study offers the unique opportunity to describe real-world patterns of drug utilization in incident cases of RA covering 87% of the German population. The objective of the study was to provide a current and nearly complete picture of the population-based frequency of drug prescriptions (DMARDs, GCs, NSAIDs) in newly diagnosed cases of RA in the ambulatory setting in Germany over the first 3 years of the disease.

## Methods

### Data source

The present study is based on all ambulatory physician billing claims and pharmacologic treatments of residents with statutory health insurance (SHI) in Germany. Because roughly 87% of residents have SHI in Germany, the present analysis can be considered a nearly total coverage of the German population. In particular, two data sources were linked for the purpose of the current study. As first source, nationwide drug prescription data from the years 2009–2015 were used comprising all ambulatory drug prescriptions that were redeemed at pharmacies by SHI-insured persons. This dataset includes information on the prescribed medication, the patient (i.e. pseudonymized patient identifier and region of residence) and the prescribing physician. As second data source, we used ambulatory physician billing claims data from all 17 regional Associations of Statutory Health Insurance Physicians (ASHIPs), covering outpatient diagnoses of all SHI-insured residents treated in ambulatory care in Germany at least once in the years 2009–2015. The ASHIPs represent the interests of the SHI-authorized physicians in ambulatory care. They verify the invoices presented by the physicians and are responsible for reimbursement from the respective statutory sickness fund. The claims dataset contains information on the patient (i.e. pseudonymized patient identifier, sex, age, region of residence) and all diagnoses related to the patient. After linkage of the two datasets based on the patient information it was possible to analyse prescription patterns with respect to the indication RA.

### Study design and study population

**Fig. 1:**
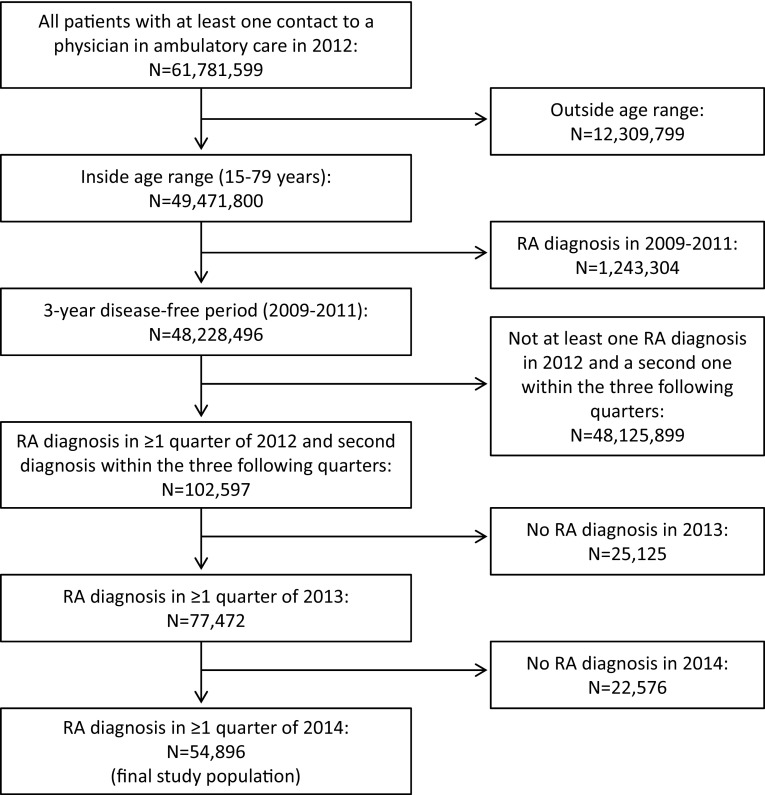
Selection of the study population

We designed a retrospective cohort study focusing on patients with newly diagnosed RA in 2012 and aged between 15 and 79 years. Figure 1 illustrates how the cohort of incident RA patients was derived from the cohort of patients with at least one physician contact in ambulatory care in 2012 (*n* = 61,781,599). RA patients were identified based on the diagnostic code (M05.-, M06.-) according to the ICD10-GM (International Statistical Classification of Diseases, German modification). Since diagnoses from ambulatory care include a diagnostic modifier describing the diagnostic certainty (“assured”, “suspected”, “status post”, “excluded”), we restricted the analysis to diagnoses that included the diagnostic certainty “assured”. A disease-free period of 3 years (2009–2011) was chosen to minimize bias due to inclusion of prevalent RA cases. Thus, incident cases of RA were defined as those patients who received their first diagnosis of RA in one quarter of the year 2012 (index quarter) and a second (confirmatory) diagnosis in one of the three quarters following the index quarter. In addition, we restricted the study population to only those patients who further had at least one RA diagnosis documented in 2013 and 2014, thereby focusing the analysis on RA patients who were in continuous ambulatory care for at least 3 years and reducing the likelihood of including false positives. The final study population comprised 54,896 patients with newly diagnosed RA in 2012 that were followed for 3 years from the index quarter.

### Medications of interest

We included prescriptions for NSAIDs, GCs, csDMARDs and bDMARDs. Table 1 lists all included agents with their respective code according to the Anatomical Therapeutic Chemical (ATC) classification system.

**Table 1:** Substances and their ATC code included in the present study

Substance	ATC code
NSAIDs	M01A
Glucocorticoids	H02AB
csDMARDs	
Auranofin	M01CB03
Chloroquine	P01BA01
Penicillamine	M01CC01
Hydroxychloroquine	P01BA02
Sulfasalazine	M01CX02
Azathioprine	L04AX01
Ciclosporin	L04AD01
Cyclophosphamide	L01AA01
Leflunomide	L04AA13
Methotrexate	L01BA01, L04AX03, M01CX01
bDMARDs	
Adalimumab	L04AB04
Certolizumab	L04AB05
Etanercept	L04AB01
Golimumab	L04AB06
Infliximab	L04AB02
Abatacept	L04AA24
Anakinra	L04AC03
Tocilizumab	L04AC07
Rituximab	L01XC02

*ATC* anatomic therapeutic chemical (ATC) classification system, *NSAIDs* non-steroidal anti-inflammatory drugs, *csDMARDs* conventional synthetic disease-modifying antirheumatic drugs, *bDMARDs* biologic disease-modifying antirheumatic drugs

### Statistical analysis

Drug prescription patterns among incident RA patients were investigated within the first, second and third year after index diagnosis and combined for the first 3 years of disease. The prescription prevalence was determined as the percentage of incident RA patients who had been prescribed the included medication in the respective time period. Prescription prevalence was studied by sex, age (< 35, 35–< 50, 50–< 65 and 65–< 80 years), RA serotype [seropositive (M05) vs. seronegative (M06)] and across specialty groups of the prescribing physician. For the analysis according to serotype, patients were considered seropositive if they had received the diagnostic code M05 at least once within the first year of disease. All other patients were regarded as seronegative. With regard to physician specialty, patients were grouped into four categories: those who had received a prescription of the included medication by (a) exclusively general practitioners and general internists (in the following GP), (b) exclusively rheumatologists, (c) GP and rheumatologists, or by (d) other specialty groups.

Associations between selected characteristics and the chance of receiving a csDMARD, a bDMARD and a GC, respectively, within the first year of disease were analysed by estimating odds ratios (OR) and 95% confidence intervals (95% CI) using logistic regression analysis. Each multivariable model included sex, age at diagnosis, RA serotype, specialty of the prescribing physician and residential region as independent variables. We also evaluated potential interaction between age and sex with regard to the chance of receiving csDMARDs, bDMARDs and GCs, respectively. *P* values for all tests of interaction were based on the likelihood ratio test for the comparison of the multivariable model with interaction term to the multivariable model without interaction term. Since all *P* values indicated significant interaction between age and sex (all *P* < 0.01), we cross-classified patients according to sex and age group and estimated ORs with 95% CIs for all subgroups in relation to women aged between 65 and < 80 years (reference).

In sensitivity analysis, we repeated the main analysis on prescription prevalence after exclusion of patients who were not prescribed any medication within the first year of disease.

## Results

We identified 54,896 patients with newly diagnosed RA in 2012, of which about one-fifth (22%) could be attributed to the seropositive type (Table 2). The majority of incident RA patients (37,352 [68%]) were women and the mean (± SD) age at diagnosis was 58.5 (14.0) years. About three quarters (74%) were 50 years and above. Half of the patients had received prescriptions only from a GP, 33% had received prescriptions from a GP and a rheumatologist and 10% had received prescriptions only from a rheumatologist.

**Table 2:** Prescription prevalence of DMARDs, GCs and NSAIDs in patients with incident RA within the first year of diagnosis

	*N*	(%)	Within first year of disease					
			DMARDs (%)			GCs (%)	NSAIDs (%)	Any prescription
			All	csDMARDs	bDMARDs			
Total	54,896	(100)	44.3	43.1	3.3	54.9	63.5	83.7
Sex								
Men	17,544	(32.0)	46.9	45.3	4.1	58.0	64.2	85.0
Women	37,352	(68.0)	43.1	42.1	2.9	53.4	63.2	83.2
Age (years)								
< 35	3854	(7.0)	52.8	48.7	10.0	47.8	60.4	82.5
≥ 35 and < 50	10,431	(19.0)	47.5	45.6	5.1	51.0	65.8	83.8
≥ 50 and < 65	20,536	(37.4)	45.7	44.8	3.1	53.1	66.4	84.3
≥ 65 and < 80	20,075	(36.6)	39.5	39.1	1.1	60.1	60.1	83.5
Serotype								
Seropositive	12,161	(22.2)	69.7	68.6	4.8	71.8	68.2	92.5
Seronegative	42,735	(77.8)	37.1	35.9	2.8	50.0	62.2	81.3
Specialty of prescribing physician^a^								
Only GP	23,081	(50.2)	36.9	36.1	2.1	56.2	79.0	100
Only rheumatologist	4726	(10.3)	68.7	64.3	8.6	63.5	49.9	100
GP and rheumatologist	14,985	(32.6)	78.9	77.7	4.9	86.1	80.6	100
Other	3182	(6.9)	23.4	20.7	4.7	39.3	69.8	100

*DMARDs* disease-modifying antirheumatic drugs, *csDMARDs* conventional synthetic DMARDs, *bDMARDs* biologic DMARDs, *GCs* glucocorticoids, *NSAIDs* non-steroidal anti-inflammatory drugs, *GP* general practitioner

^a^Only patients with any prescription within the first year of disease were included (*N* = 45,974)

**Fig. 2:**
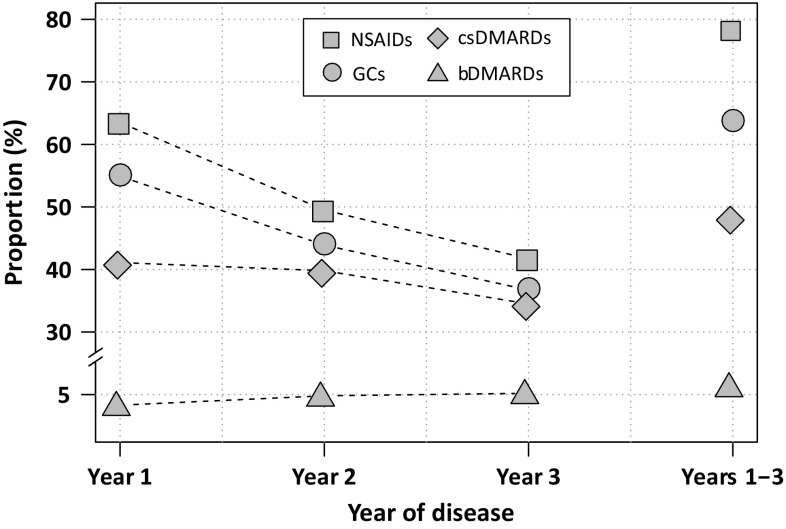
Prescription prevalence of medications of interest within the first 3 years of RA disease. *csDMARDs* conventional synthetic disease-modifying antirheumatic drugs, *bDMARDs* biologic disease-modifying antirheumatic drugs, *GCs* glucocorticoids, *NSAIDs* non-steroidal anti-inflammatory drugs

Approximately 44% of the incident RA patients received a DMARD within the first year of RA disease (Table 2). Specifically, 41% received only a csDMARD, 2.1% received a csDMARD and a bDMARD and 1.2% received solely a bDMARD. A total of 3.3% was prescribed a bDMARD within the first year of disease. About 55% and 64% of patients received GCs and NSAIDs, respectively, in the first year of disease. Among those patients without DMARD prescription, the majority (70%) was treated with GCs and/or NSAIDs (11% received only GCs, 24% NSAIDs and GCs, and 35% received only NSAIDs). With increasing duration of disease, the prescription prevalence of csDMARDs and GCs decreased, while bDMARDs were prescribed more frequently (Fig. 2). Within the first 3 years of RA disease, 64%, 48% and 6.3% patients had received a prescription of GCs, csDMARDs and bDMARDs, respectively.

The prescription prevalence of all investigated medications was higher among males compared to females and varied according to age. Younger patients were more likely to receive DMARDs and less likely to receive GCs than their older counterparts. Specifically, 49% and 48% of patients aged < 35 years received a csDMARD and a GC, respectively, while 40% and 60% of patients aged ≥ 65 years were prescribed a csDMARD and a GC, respectively. The age-related difference in prescription frequency was particularly pronounced for bDMARDs: while 10% of RA patients aged < 35 years received bDMARDs, only 1.1% of those aged ≥ 65 years received bDMARDs. The prescription prevalence of any DMARD was twofold higher in patients diagnosed with seropositive RA vs. patients diagnosed with seronegative RA (70% vs. 37%). Similarly, patients who had received prescriptions from both, a GP and a rheumatologist, were twice as likely to receive DMARDs as patients who only had prescriptions from GP (79% vs. 37%).

**Fig. 3:**
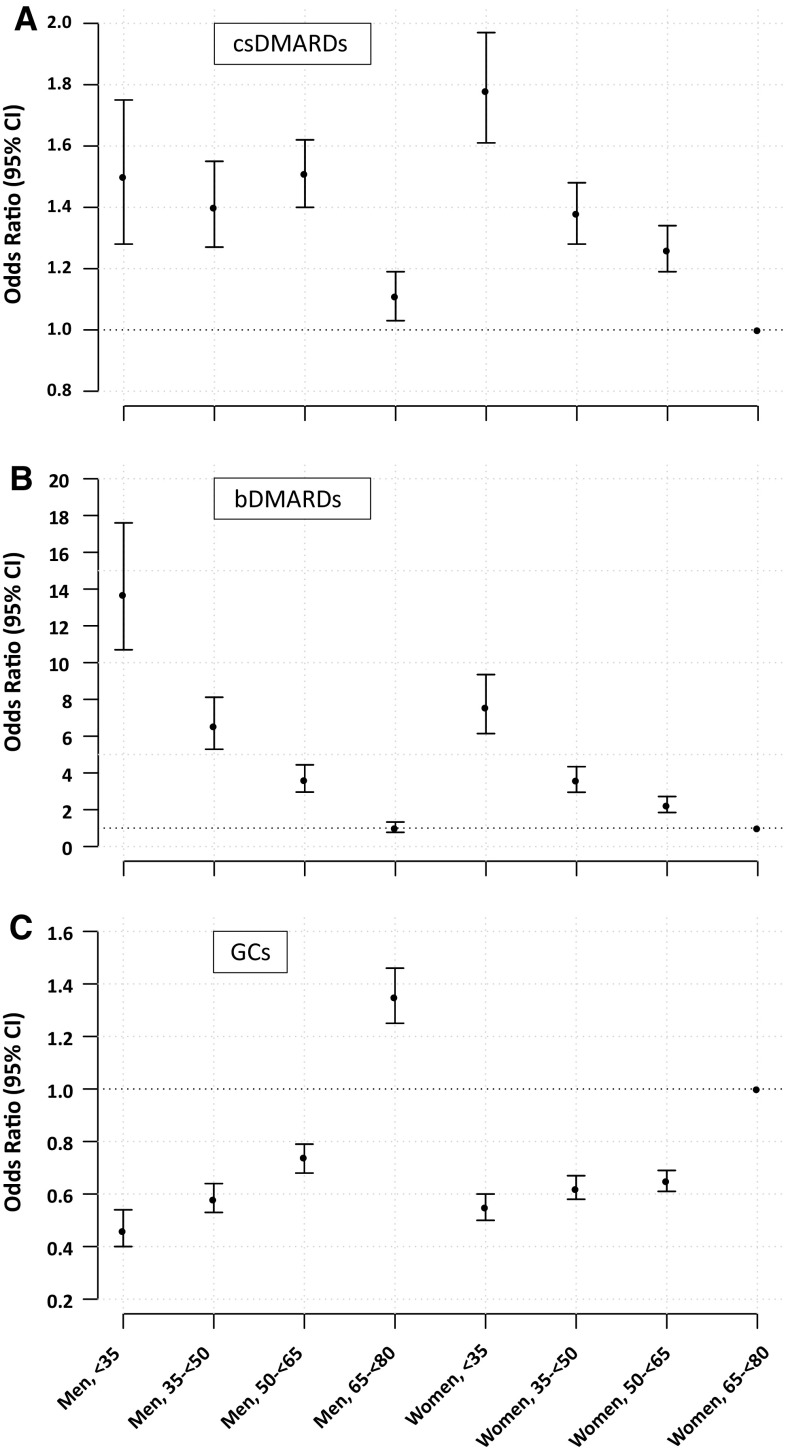
Multivariable-adjusted Odds Ratio (95% CI) for the chance of receiving csDMARDs (**a**), bDMARDs (**b**) and GCs (**c**) within the first year of disease according to sex and age group with women aged between 65 and < 80 as reference. Estimates of effect originate from three separate logistic regression models containing groups of age and sex and adjustment for serotype, specialty of prescribing physician and residential region. Only patients with any prescription within the first year of disease were included (*N* = 45,974). *csDMARDs* conventional synthetic DMARDs, *bDMARDs* biologic DMARDs, *GCs* glucocorticoids

All bivariate associations persisted in the multivariable logistic regression model (Table 3). Women had consistently and significantly lower odds of receiving a csDMARD, bDMARD and GC within the first year of disease compared to men. Specifically, the ORs (95% CI) for women receiving prescriptions for csDMARDs, bDMARDs and GCs were 0.90 (0.87–0.95), 0.62 (0.57–0.69) and 0.88 (0.84–0.92), respectively. Younger patients (< 35 years) were 60% more likely to receive a csDMARD but 50% less likely to receive a GC within the first year of disease compared to their older counterparts (≥ 65 years). With respect to bDMARDs, young patients had almost tenfold higher chances of receiving a prescription compared to patients aged ≥ 65 years [OR (95% CI) = 9.5 (8.0–11.3)]. The cross-classification of sex and age group resulted in a differentiated picture on the interaction between sex and age with regard to the receipt of csDMARDs, bDMARDs and GCs (Fig. 3a–c). In terms of csDMARDs, the association of age differed noticeably between sexes (Fig. 3a). While among men, the chances of receiving a csDMARD did not differ between patients up to the age of 65, among women a strong linear inverse association across age groups was observed, demonstrating that the lower odds observed for the group of all women in the multiple logistic regression model as described above was driven by the middle-aged and older women (50–< 65 years and ≥ 65 years). Young women (< 35 years) had the highest chances of early csDMARD receipt compared to all cross-classified subgroups [OR (95% CI) = 1.78 (1.61–1.97)]. With regard to bDMARDs, we observed a strong inverse association with age, both among men and women, though men had significantly higher chances of receiving bDMARDs up to age of 65 years compared to women (Fig. 3b). With regard to GCs, differences in the chance of a prescription were less pronounced between men and women below the age of 65, while older men were 35% more like to receive GCs than women at the same age [OR (95% CI) = 1.35 (1.25–1.46)].

**Table 3:** Multivariable-adjusted odds ratio (95% CI) for the association of demographics, RA serotype and prescribing physician with prescriptions of DMARDs and GCs within the first year of disease

	csDMARDs	bDMARDs	Glucocorticoids
Women vs. men	0.90 (0.87–0.95)	0.62 (0.57–0.69)	0.88 (0.84–0.92)
Age (years)			
< 35	1.65 (1.51–1.80)	9.5 (8.0–11.3)	0.48 (0.44–0.52)
≥ 35 and < 50	1.34 (1.27–1.43)	4.56 (3.89–5.34)	0.56 (0.52–0.59)
≥ 50 and < 65	1.29 (1.23–1.36)	2.71 (2.34–3.16)	0.61 (0.59–0.64)
≥ 65 and < 80 (Ref.)	1.00	1.00	1.00
Seropositive vs. seronegative	2.77 (2.63–2.91)	1.32 (1.18–1.46)	1.70 (1.60–1.80)
Specialty of prescribing physician			
Only GP (ref.)	1.00	1.00	1.00
Only rheumatologist	3.12 (2.91–3.33)	3.83 (3.33–4.41)	1.45 (1.36–1.55)
GP and rheumatologist	5.89 (5.61–6.19)	2.24 (1.99–2.53)	4.77 (4.52–5.04)
Other	0.43 (0.39–0.47)	2.04 (1.68–2.47)	0.53 (0.49–0.57)

Estimates of effect originate from three separate logistic regression models containing all listed variables and additional adjustment for residential region. Only patients with any prescription within the first year of disease were included (*N* = 45,974)

*DMARDs* disease-modifying antirheumatic drugs, *csDMARDs* conventional synthetic DMARDs, *bDMARDs* biologic DMARDs, *GCs* glucocorticoids, *GP* general practitioner

In 71% of the cases, the initial DMARD prescribed was methotrexate, followed by sulfasalazine (12%) and hydroxychloroquine (8%). In 5.6% of the cases, more than one DMARD was prescribed in the quarter of the index diagnosis, mainly including other csDMARDs in addition to methotrexate. The most important bDMARDs as initial agents were adalimumab (2.1%) and etanercept (1.6%). In terms of the whole first year of disease, 75% of incident patients who had been prescribed a DMARD received methotrexate, 14% sulfasalazine, 11% leflunomid and 10% hydroxychloroquine.

After excluding patients who did not receive any medication during the first year of disease, the prescription prevalence of total DMARDs, csDMARDs, bDMARDs, GCs and NSAIDS was 53%, 52%, 3.9%, 66% and 77%, respectively.

## Discussion

In a real-world setting comprising data of 87% of the German population, we evaluated patterns of drug prescriptions among patients with newly diagnosed RA in ambulatory care. The present study thereby adds to the latest knowledge on how guideline recommendations on DMARD treatment in newly diagnosed RA have been translated into actual clinical practice in Germany. Less than half of the incident RA cases received a DMARD within the first year of disease and substantial differences were observed according to age, sex, RA serotype and specialty of prescribing physician.

It is undoubted that the appropriate and timely use of DMARDs can improve clinical and long-term outcomes in RA [17, 18]. Hence, the importance of initiating DMARD therapy as soon as possible after diagnosis of RA is consistently emphasized as the mainstay of modern RA management in national and international guidelines [6, 7, 9–11, 19]. Our observation indicates that a substantial proportion of patients do not receive DMARDs within the first year of disease suggesting a general underuse of DMARDs in treatment of newly diagnosed RA. Reasons underlying this observation remain unresolved so far but may comprise (a) contraindications to DMARD therapy, (b) a very mild course of the disease initially not requiring DMARD treatment and (c) doctors’ or patient attitudes. In case of mild disease, low disease activity and no indication of progression, patients may initially refrain from DMARD therapy according to guidelines [20]. In line with this, we found that the majority of patients without DMARD use were treated with GCs and/or NSAIDs. Bukhari et al. observed that roughly 30% of RA patients have a mild disease course with hardly any progression in radiographic outcome over 5 years [21]. Further, the attitudes of doctors’ and patients’ towards aggressive therapy, potential side-effects and route of administration may impact utilization of DMARDs [1, 22].

Most strikingly, we observed considerable disparities in the initiation of DMARD therapy across patient subgroups implying an inequitable access among patients with newly diagnosed RA. The German health care system is committed to providing universal and equitable access to high-quality medical services for all residents. Therefore, medical treatment would be primarily driven by need factors while predisposing factors, including age and sex, or enabling factors (i.e. visit to a rheumatologist) should not determine access to treatment.

In the present study, women generally had lower chances of receiving DMARDs and GCs compared to men. This observation was almost consistently present across all age groups, suggesting that women may be systematically disadvantaged with regard to early initiation of adequate RA therapy. In terms of csDMARDs, we demonstrated a complex interplay of sex with age and identified old women (aged ≥ 65 years) as the most vulnerable patient group with the lowest chance of receiving a csDMARD compared to all other subgroups of age and sex. Conversely, young women had the highest chance of being prescribed a csDMARD, highlighting considerable inequities within the group of women. To our knowledge, a differential effect of age among men and women has not been described before and reasons for this finding remain to be elucidated. There is evidence to suggest that women’s access to adequate RA therapy may have improved during the first decade of the twenty-first century [23]. The authors of a Norwegian study hypothesized that this may be due to the increased women’s ability to communicate RA-related limitations and needs as a result of continuously disappearing gender differences in society within recent decades. Since we did not observe a differential association between sex and chance of csDMARD therapy among patients younger than 50 years, the finding that women aged ≥ 50 years were disadvantaged could, therefore, at least partly, reflect traditional gender role attitudes and may essentially represent a cohort effect. Given that Germany lags behind Norway with regard to equality of sexes, the observed sex difference might continuously decrease over the next years. Nonetheless, future research may undertake efforts to elucidate gender differences in uptake of drugs in RA therapy.

With regard to age, our study suggests a preferential treatment of older RA patients with glucocorticoids rather than DMARDs. Discrepancies in receipt of DMARDs according to age have already been observed in earlier population-based studies [16, 24–30], though reasons for this age-related variation are currently unknown. Tutuncu et al. reported this age difference to be independent of duration, severity and activity of the disease [27]. A survey among US rheumatologists revealed that rheumatologists’ treatment recommendations may be influenced by the patient’s age with a preference for aggressive treatment among younger compared to older patients given the same disease activity and comorbidities [31]. Concerns regarding a differential toxicity of drugs among older persons, including a higher risk for infections, or patient-related factors such as lower health literacy, comorbidities, contraindications and fear of initially trying an “aggressive” treatment with potentially considerable side effects may partly explain the differential prescribing of DMARDs according to age [27, 30, 32]. However, with regard to bDMARDs, inequalities by age remained unchanged after controlling for the number of comorbidities in a Norwegian study [30]. Given that more than one-third of RA patients is diagnosed at ≥ 65 years of age and studies generally show older age at onset to be related to higher disease activity [33], future studies may further examine the factors influencing the choice of pharmacotherapy in older persons with new-onset RA in more detail, also with focus on gender differences. In addition, efforts to increase the physicians’ awareness of this potential age bias in RA treatment may be useful to ensure equal health care delivery across age. Furthermore, research in accurate RA medication in the context of multi-morbidity and polypharmacy, especially in the elderly, should be addressed and translated into future guidelines.

The present study emphasizes the key role of rheumatologists for the appropriate care of RA, which is in line with previous studies [12, 16, 34]. It is noteworthy though, that only 43% of incident RA patients in this large population-based dataset had contact with a rheumatologist based on the receipt of a prescription within the first year of disease. This finding is consistent with the recent study of Albrecht et al. who found that 40% of the prevalent cases identified based on claims data from a German statutory health insurance fund had at least one contact with a rheumatologist in the ambulatory setting within the study period of 1 year [16]. In reality, the proportion of patients seen by a rheumatologist may be a little higher since we were not able to identify those additional patients with ambulatory treatments in specialty care conducted in university ambulances and by rheumatologists working as general practitioners. Based on data from the German Collaborative Arthritis Centers and results from a small population survey involving RA patients, it is assumed that about 60% of patients are in rheumatological specialty care in Germany [35, 36]. Considering the differential prescription prevalence according to specialty of the physician, an improved rapid referral to rheumatologists indicates a considerable potential for optimizing care of RA patients. However, to cover the corresponding demand the German Society for Rheumatology recently estimated that twice as many rheumatologists would be needed for ambulatory specialty care as currently exist [37].

We further found an almost threefold higher chance of receiving a csDMARD within the first year of disease in seropositive compared to seronegative patients, which was independent of age, sex, specialty of the prescribing physician and region of residence. Similarly, in a French multicenter cohort study, the presence of seropositivity was related to higher conformity to treatment guidelines in the multivariable analysis in comparison to seronegativity [38]. Since the presence of seropositivity is generally related to more severe symptoms and joint damage [1], a more intense treatment in seropositive patients would be expected. Nevertheless, a German population survey reported considerable deficits in the treatment of seronegative patients upon clinical examination [36]. In that study, 46% of seronegative cases were considered to be insufficiently treated. Likewise, in the German Collaborative Arthritis Centers, seronegative patients frequently report an impaired quality of life and often receive analgesics [15]. Thus, an unmet need for DMARDs may exist in this subgroup and further studies may focus on reasons for these marked differences in treatment according to serotype.

Among the strengths of the present study are the real-life setting and the large sample size covering 87% of the German population. In addition, these data allowed for the complete ascertainment of dispensed ambulatory prescriptions of DMARDs in an unselected population. Finally, the length of the study period (2009–2015) allowed for a rigorous case definition including a disease-free period of 3 years and a 2-year confirmation period with continuous documentation of RA diagnosis. Limitations of our study refer to the commonly recognized constraints of administrative claims data. First, the inclusion of patients was based on the presence of specific diagnostic codes and was, therefore, dependent on the accuracy of these codes. We addressed this limitation by applying a strict case definition requiring documentation of RA diagnosis in several years to ensure a high specificity. Misdiagnosis of RA may exist particularly in the early stages of the disease and we cannot entirely rule out that some of the included patients actually do not have RA. Including false positive cases in our analysis would have resulted in a lower prescription prevalence than actually present. Nevertheless, the prescription frequency of DMARDs increased only moderately when we restricted the study population to patients with at least one dispensed prescription within the first year of disease, still implying a considerable underuse. Second, administrative claims data do not allow determining disease activity/severity which, however, is central to therapeutic decisions in RA. The lack of this information has to be kept in mind when interpreting our results as real-world evidence of the adoption of clinical treatment recommendations. Third, although we tried to account for confounding factors in the multivariable regression model, we were not able to account for variables not available in the administrative data base, including socioeconomic status, smoking and clinical parameters, which may be related to the initiation of DMARDs. Fourth, since our data were limited to drugs that were redeemed at pharmacies, we may underestimate the use of NSAIDs which are frequently purchased directly over-the-counter. Finally, our analysis was restricted to prescriptions from ambulatory care and did not include medications administered in hospitals. Despite that, it is unlikely that we underestimated prescription prevalence of DMARDs since patients are only treated in hospital for a short time period and any DMARD therapy that is initiated in hospital will be continued in ambulatory care and thus included in the present dataset.

## Conclusion

Despite disease modification is the mainstay of modern RA treatment, this nationwide population-based study revealed that in the majority of patients DMARD therapy is not initiated within the first year of disease. This suggests that further efforts may be needed to ensure full implementation of recommendations in clinical practice. Specialized rheumatology care emerged as a key factor for adequate treatment. Our study further identified older and seronegative patients as particularly vulnerable groups with lower likelihood of receiving DMARDs. Future studies may investigate  the factors underlying these inequalities in DMARD prescriptions.
